# Bridging Imaging and Pathohistology in Pancreatic Hamartoma: A Systematic Review of the Literature with an Integrated Case Report

**DOI:** 10.3390/jcm15010136

**Published:** 2025-12-24

**Authors:** Dunja Stankic, Nina Rajovic, Nikola Grubor, Jelena Rakocevic, Aleksandar Ninic, Marjan Micev, Jelena Vladicic Masic, Luka Joksimovic, Natasa Milic, Kristina Davidovic, Nikica Grubor

**Affiliations:** 1 Department for HBP Surgery, Clinic for Digestive Surgery, University Clinical Centre of Serbia, 11000 Belgrade, Serbia; dunjast@gmail.com (D.S.);; 2 Institute for Medical Statistics and Informatics, Faculty of Medicine University of Belgrade, 11000 Belgrade, Serbia; nikola.n.grubor@med.bg.ac.rs (N.G.);; 3 Department of Radiology, Clinical Center of Serbia, 11000 Belgrade, Serbia; 4 Institute of Histology and Embryology “Aleksandar Đ. Kostić”, Faculty of Medicine, University of Belgrade, 11000 Belgrade, Serbia; 5 Department for Pathology, Clinic for Digestive Surgery, University Clinical Centre of Serbia, Koste Todorovića 6, 11000 Belgrade, Serbia; 6 Department of Internal Medicine, Faculty of Medicine, University of East Sarajevo, 73300 Foca, Bosnia and Herzegovina

**Keywords:** pancreatic hamartoma, case report, systematic review, radiography, MRI, CT, histological pattern

## Abstract

**Background:** Pancreatic hamartoma (PH) is an exceptionally rare, benign, mass-forming lesion accounting for less than 1% of all pancreatic tumors. Its rarity and non-neoplastic nature contribute to significant diagnostic challenges, often leading to misclassification as malignant disease. This study presents a case of PH and a systematic review of all reported cases, with emphasis on histopathological and imaging characteristics. **Methods:** A comprehensive electronic search of PubMed, Scopus, and Web of Science was conducted up to 1 April 2025, to identify eligible case reports and series. **Results:** We describe a 37-year-old woman with a cystic lesion of the pancreatic tail, ultimately confirmed histologically as a cystic pancreatic hamartoma following distal pancreatectomy with splenectomy, with an uneventful postoperative course. Of 687 screened studies, 51 met the inclusion criteria, comprising 77 cases (68 adults, 9 pediatric). PHs occurred most frequently in males (52.9%), with a mean age of 59.5 ± 12.9 years, and were often asymptomatic (57.4%). The pancreatic head was the most common site (52.9%). On MRI, PHs typically exhibited low T1-weighted and high T2-weighted signal intensity, with no FDG uptake (82%) and moderate or no restriction on DWI, distinguishing them from neuroendocrine tumors (NETs). Histologically, most lesions were solid (64.7%) or solid–cystic (35.3%), with low spindle cell cellularity and absent Langerhans islets. **Conclusions:** Low T1WI signal and moderate DWI signal are the key features distinguishing PHs from NETs. Incorporating these findings with EUS-FNA and immunohistochemistry can support a provisional diagnosis and help avoid unnecessary radical surgery.

## 1. Introduction

Hamartomas are benign neoplasms characterized by the disorganized proliferation of normal cells and tissues native to the site of origin [1]. Although their exact global prevalence remains unknown, this is likely attributable to the rarity of these lesions [2]. Among hamartomas, pancreatic hamartoma represents an exceptionally rare, benign, mass-forming lesion that accounts for less than 1% of all pancreatic neoplasms. Its non-neoplastic nature and infrequency contribute to the considerable challenges associated with accurate preoperative diagnosis [3].

Pancreatic hamartoma was first described by Anthony et al. in 1977 [4]. Subsequently, Pauser et al. and Yamaguchi et al. proposed pathological criteria to define this rare entity, which include: the presence of a well-circumscribed mass, a composition of mature acini and ducts exhibiting disordered architecture and an absence of identifiable islets of Langerhans [5,6,7]. Based on these features, pancreatic hamartomas are further classified into two subtypes: solid or solid and cystic forms [8].

Despite advances in diagnostic imaging technologies, differentiating benign pancreatic tumors, such as hamartomas, from malignant lesions remains a significant clinical challenge. The increasing use of modalities including computed tomography (CT), magnetic resonance imaging (MRI), endoscopic retrograde cholangiopancreatography (ERCP), and particularly endoscopic ultrasound (EUS) has led to more frequent detection of asymptomatic pancreatic lesions [9,10]. However, pancreatic hamartomas lack definitive radiological features, and tissue samples obtained through fine needle aspiration (FNA) are often insufficient for establishing a reliable pathological diagnosis [11]. Consequently, these lesions are frequently misclassified as other pancreatic tumors, leading to surgical resection to achieve a definitive diagnosis [12], which typically relies on histopathological and immunohistochemical evaluation [10]. Although radiological and pathological reports on pancreatic hamartomas have become more numerous in recent years, and imaging features have been increasingly clarified [13,14], a precise correlation between radiological and pathological findings has yet to be established.

Here, we present a case report of pancreatic hamartoma and provide a systematic review of all available reported cases, with a particular focus on differences between histopathological and imaging findings, contributing valuable insights to the existing body of knowledge on this rare entity.

## 2. Materials and Methods

The systematic review was conducted in compliance with The CARE guidelines (for CAse REports) [15] and Preferred Reporting Items for Systematic Reviews and Meta-Analyses (PRISMA) [16]. The review methodologies were predetermined, and no substantial deviations from the established protocol were noted. The review has been formally registered in the Open Science Framework (OSF) (registration DOI: 10.17605/OSF.IO/HM9UN).

### 2.1. Selection of Studies

The study inclusion screening process for the systematic review was carried out in two phases. At every phase, two reviewers independently evaluated the studies, resolving discrepancies through discussion or consensus. A third reviewer was engaged to address any remaining disagreements. Eligible studies comprised published case reports concerning pancreatic hamartoma. The exclusion criteria included: (1) populations other than those with pancreatic hamartoma, (2) studies not categorized as case reports or case series (reviews), (3) inappropriate population (non-human subjects), and (4) publications not in English.

### 2.2. Search Strategy

The search strategy was collaboratively designed by two reviewers, one having expertise in abdominal surgery and the other competent in search strategy design. A comprehensive electronic search of PubMed, Scopus, and Web of Science was performed until April 1st, 2025, to identify published studies containing the term “pancreatic hamartoma” OR “pancreas hamartoma”. Detailed search strategies, including the exact search strings used for each database, are available in the Supplementary Materials.

### 2.3. Data Abstraction

Data extraction was performed independently by two reviewers and encompassed the following variables: study title, author(s), year of publication, country of origin, patient demographics (gender and age), clinical presentation (asymptomatic or symptomatic), duration of symptoms, tumor location and size, reported instances of communication and pancreatitis, imaging techniques utilized, performance of endoscopic ultrasound-guided fine-needle aspiration (EUS-FNA), provisional diagnoses, treatment modalities, histological characteristics, immunohistochemical results, evidence of recurrence and metastasis, documented postoperative complications, and duration of follow-up. Authors of relevant articles were contacted to obtain missing data. Data synthesis and visual presentation were performed using structured summary tables summarizing key demographic, clinical, imaging, and pathological variables.

### 2.4. Statistical Analysis

Numerical data were presented as mean or median with corresponding measures of variability (standard deviation, IQR). Categorical variables were summarized by absolute numbers with percentages. Fisher’s exact test was used to assess the differences between radiological characteristics and pathological findings. A *p*-value of <0.05 was considered to be statistically significant. Analysis was performed using SPSS for Windows (21.0; IBM SPSS, Chicago, IL, USA).

## 3. Results

### 3.1. Case Presentation

A 37-year-old female patient underwent treatment for a suspicious lesion on the thigh, highly suggestive of melanoma. Following radical surgical excision of the lesion, additional diagnostic evaluations were performed, including an abdominal ultrasound. This imaging revealed an abnormality abutting the splenic vein in the splenic hilum, initially characterized as a cyst of the pancreatic tail. The patient denied weight loss, nausea, or vomiting but reported intermittent sharp pain beneath the left rib cage, radiating to the back, particularly pronounced after meals. This pain had been present for several years. Physical examination revealed no pathological findings or palpable masses.

The patient’s medical history included endoscopic polypectomy of the uterine cavity in 2017. Hematological testing showed heterozygosity for prothrombin gene mutation (Factor II), for which no specific medical intervention was required. Laboratory analyses showed that tumor markers, including CA 19-9 (33 kU/L), alpha-fetoprotein (AFP), carcinoembryonic antigen (CEA), CA 125, and CA 72-4, were within reference ranges. The complete blood count and biochemical profile were also unremarkable.

#### 3.1.1. Imaging Findings

Subsequent imaging included abdominal ultrasound and computed tomography (CT), which identified a mesenteric cyst adjacent to the anterior aspect of the pancreatic tail, initially suggesting it was not arising from the pancreas. Multiple magnetic resonance imaging (MRI) and magnetic resonance cholangiopancreatography (MRCP) studies, performed over a six-month period, demonstrated a bilocular cystic lesion measuring 20 × 27 × 29 mm, differentiating two compartments with a wall thickness of up to 3 mm. Preoperative imaging noted a subtle internal septum without a clearly visible mural nodule, and mild post-contrast enhancement of both the cyst wall and septum. The lesion exhibited partial extrapancreatic extension and lacked a defined connection with the main pancreatic duct, which remained of normal caliber.

Additionally, an encapsulated multilocular cystic formation measuring 13 × 11 × 30 mm was identified, along the anterior surface of the spleen. A CT scan with a pancreatic protocol suggested a high likelihood of mucinous cystic neoplasm (MCN), with a probable diagnosis of mucinous cystadenoma.

#### 3.1.2. Surgical Findings

The patient underwent surgery via a left subcostal laparotomy. Intraoperative exploration revealed no pathological changes in the liver or peritoneum, and no free fluid was observed in the abdominal cavity. Upon entering the omental bursa, inspection of the pancreas identified a neoplastic lesion measuring 4 × 3 cm in the pancreatic tail, which was adherent to both the splenic flexure of the colon and the splenic hilum. The cystic formation located along the anterior surface of the spleen, as described on imaging, was not confirmed intraoperatively as a distinct pathological entity. The procedure began with mobilization of the inferior margin of the pancreas and dissection of the tumor from the colon. The spleen was then mobilized, and the superior pancreatic margin was freed. The splenic artery was isolated and ligated, followed by clamping of the splenic vein. The pancreas was transected, completing the distal pancreatectomy with splenectomy. The main pancreatic duct, which measured approximately 1 mm in diameter and showed no evidence of dilation, was individually sutured. The pancreatic resection margin was reinforced with fibrin glue.

Following resection, intraoperative hemostasis was evaluated using rotational thromboelastometry (ROTEM). In view of a hypercoagulable profile, the patient received tranexamic acid along with oral antiplatelet and anticoagulant therapy. The postoperative course was uneventful, and the patient was discharged home on postoperative day eight. The patient has been compliant with routine follow-up evaluations and remained alive and well at four years of follow-up.

#### 3.1.3. Gross Pathological Findings

**Figure 1:**
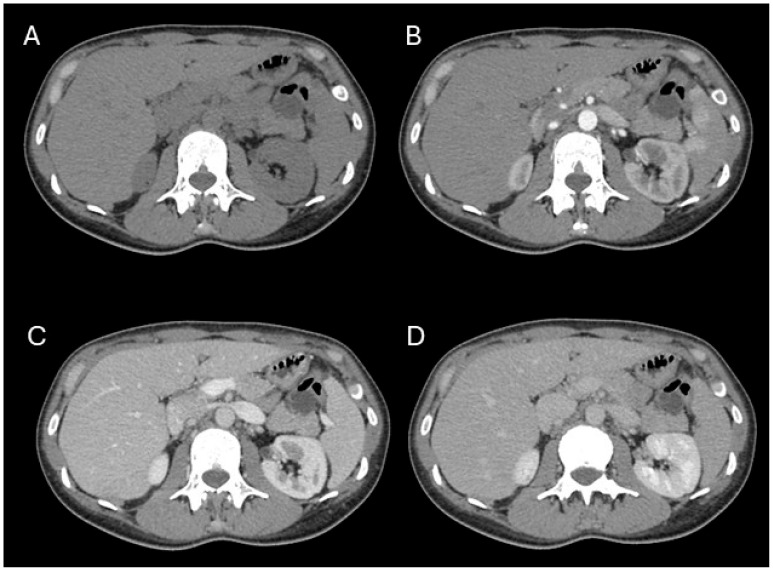
Axial contrast-enhanced CT images of the abdomen demonstrating a well-defined, lobulated cystic lesion in the pancreatic tail, consistent with a cystic pancreatic hamartoma. The lesion is visible across multiple phases without evidence of solid enhancing components. (**A**) Pre-contrast phase. (**B**) Arterial phase. (**C**) Portal venous phase. (**D**) Delayed phase. The lesion remains hypoattenuating relative to the enhancing pancreatic parenchyma throughout all phases, with no communication with the main pancreatic duct or surrounding infiltration.

**Figure 2:**
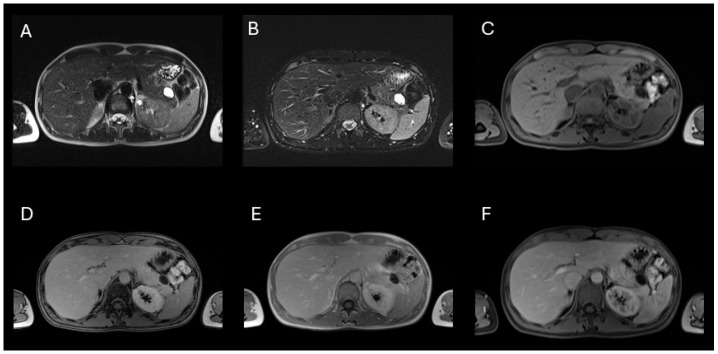
Axial MRI of the abdomen demonstrating a well-circumscribed, multiloculated cystic lesion in the pancreatic tail, consistent with a cystic pancreatic hamartoma. (**A**) T2-weighted HASTE image shows a hyperintense lesion with internal septations. (**B**) T2-weighted BLADE image confirms fluid content and sharper margin delineation. (**C**) Axial T1-weighted VIBE image pre-contrast shows a hypointense lesion. (**D**,**E**) In-phase and out-of-phase images reveal no signal drop out, excluding intralesional fat. (**F**) Portal venous phase post-contrast T1-weighted VIBE image shows no appreciable enhancement of the lesion, with preserved background pancreatic enhancement. The imaging findings are consistent with a benign cystic lesion, and no features suggest malignancy or ductal communication.

**Figure 3:**
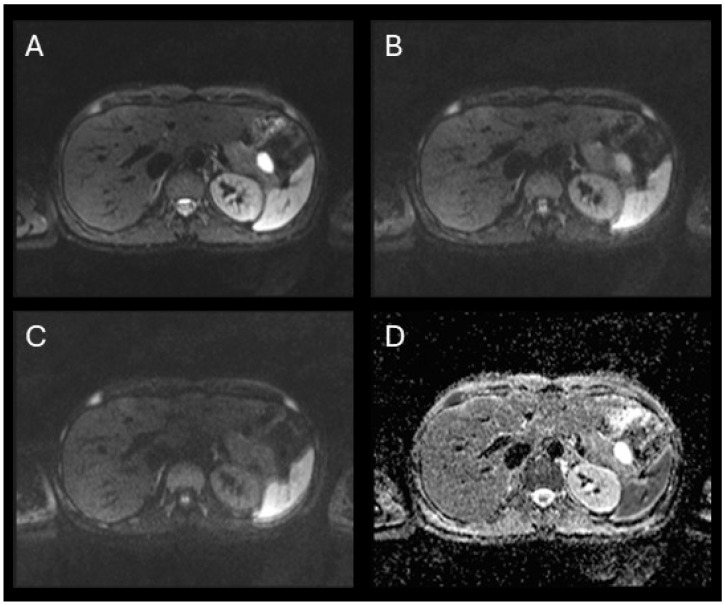
Axial diffusion-weighted MRI of the abdomen evaluating the cystic lesion in the pancreatic tail. (**A**–**C**) DWI images at b-values of 50 (**A**), 400 (**B**), and 800 s/mm^2^ (**C**) show progressive signal loss with increasing b-values. The lesion demonstrates high signal on b50 due to T2 shine-through, with marked signal attenuation by b800, indicating free diffusion. (**D**) The corresponding ADC map shows high signal intensity within the lesion, confirming the absence of restricted diffusion. These findings are consistent with a benign cystic lesion, such as cystic pancreatic hamartoma.

Gross examination of the pathological specimen revealed a well-circumscribed, mixed cystic solid lesion measuring 40 × 30 × 30 mm in the pancreatic tail. The specimen had a smooth external surface that partially extended over the anterior aspect of the gland with interspersed irregular patchy fatty tissue of approximately 7 × 5 mm in size. Sectioning of the specimen demonstrated two distinct cystic locules: one filled with a sero-mucinous fluid and the other containing a gray-yellow mucinous material. These cysts were sharply demarcated from the surrounding pancreatic parenchyma and showed expansile growth without involving the main pancreatic duct. Between the two locules lay a 10 × 10 × 5 mm solid mural nodule, which on the cut section appeared light gray-pink, moderately firm, and finely fibrous (Figure 1, Figure 2 and Figure 3).

#### 3.1.4. Pathohistological Findings

**Figure 4:**
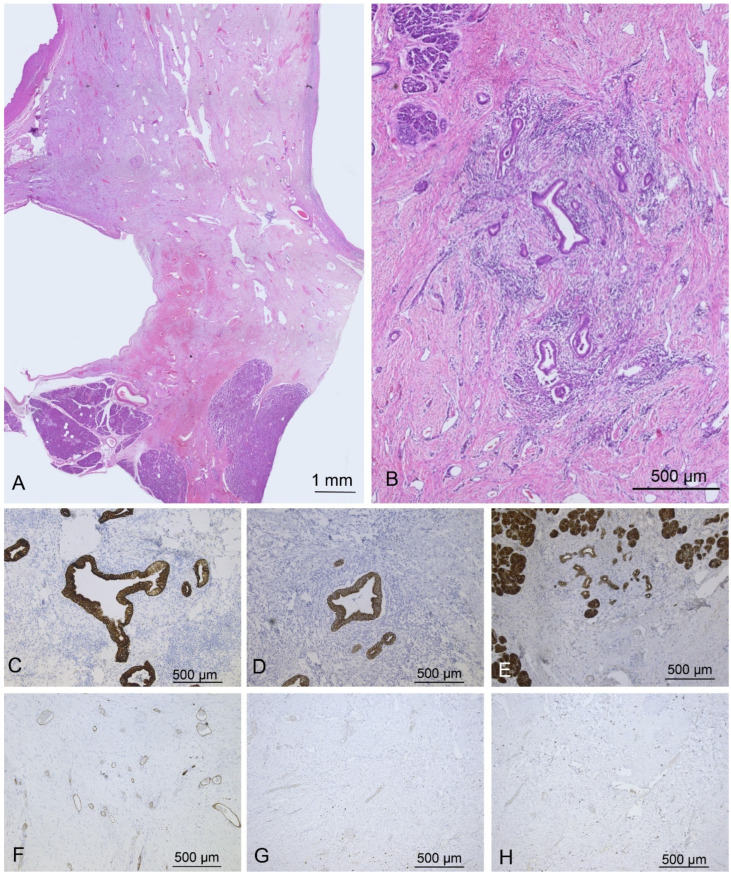
(**A**) Histology of the hamartoma revealed a solid component with prominent spindle cell mesenchymal, vascular and neuronal proliferation between cystic lesions. Cystic component showed ductal cuboid and flattened epithelium without cellular atypia; (**B**) On closer examination, some areas of ductal hyperplasia surrounded by mesenchymal cells were observed. The ductal elements showed CK7 (**C**), CK19 (**D**), and MUC1 immunopositivity (**E**), along with CK34 (**F**) and CD117 (**G**) immunonegativity. A low proliferative index was detected by Ki-67 staining (**H**).

Microscopically, the lesion exhibited heterogeneous proliferation of mesenchymal and epithelial elements. The mesenchymal component predominated and consisted of spindle-shaped cells arranged in subtle swirling and partial fascicles within an edematous, myxoid stroma. Vascular proliferation was prominent, comprising numerous dilated capillaries and occasional venules, alongside irregular nerve bundles. The epithelial component formed cystic structures lined by a single layer of flattened, cuboidal, or, less commonly, columnar cells. Mucin production by these epithelial cells was infrequent, and occasional desquamation was noted. Peripheral pancreatic ductules exhibited areas of low-grade pancreatic intraepithelial neoplasia (PanIN I), with only one microfocus of similar dysplasia within the cystic regions; no evidence of neoplastic epithelial proliferation was present (Figure 4).

Immunohistochemical staining of the epithelial component demonstrated strong positivity for pancytokeratin AE1/AE3, CK7, CK19, CAM5.2, and MUC1, with focal expression of MUC5AC. Desmin and α-SMA highlighted concentric muscle bundles adjacent to the larger cysts, whereas the remaining stromal cells were vimentin positive. The epithelium was negative for CK20, mCEA, and MUC4, and the mesenchymal component did not express CD117, CD34, α-SMA, desmin, or S 100 protein. A focal Ki 67 proliferation index of approximately 3% was observed. These combined histopathological and immunohistochemical findings supported a diagnosis of solid cystic pancreatic hamartoma.

### 3.2. Systematic Review

**Figure 5:**
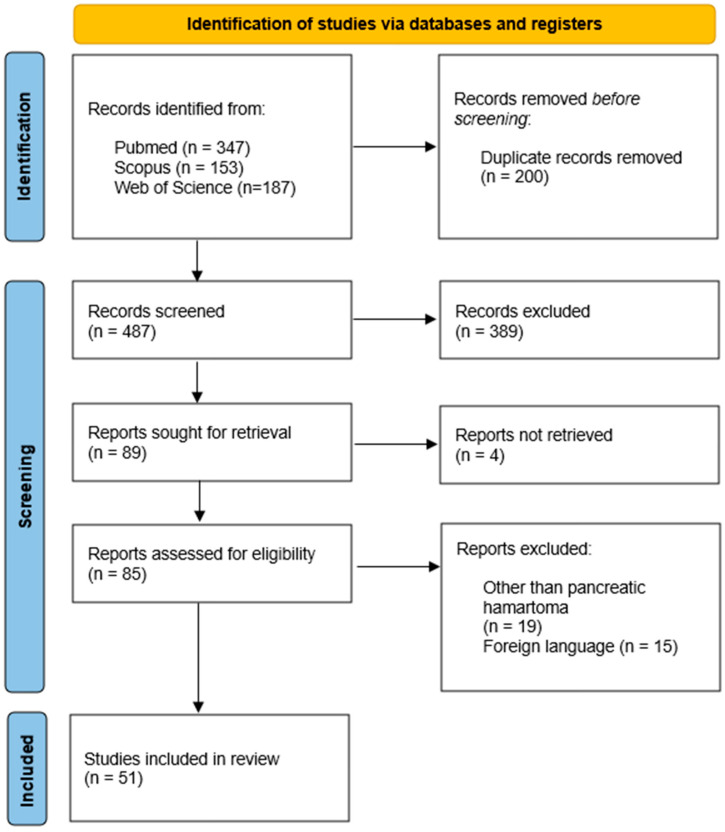
Flowchart of study selection process.

A total of 687 potentially eligible case reports were identified from four electronic databases. Following the elimination of duplicates, 487 titles and abstracts were evaluated for relevance. Out of these, 389 reports failed to satisfy the eligibility criteria, resulting in 89 case reports available for further evaluation. Due to the inability to retrieve four reports, 85 reports underwent a comprehensive evaluation. After this screening, 15 studies were excluded due to foreign language, and 19 for not involving pancreatic hamartoma. A total of 51 studies were chosen for review. The process of study selection is illustrated in Figure 5, in accordance with the PRISMA flow diagram.

### 3.3. Characteristics of Eligible Studies

**Figure 6:**
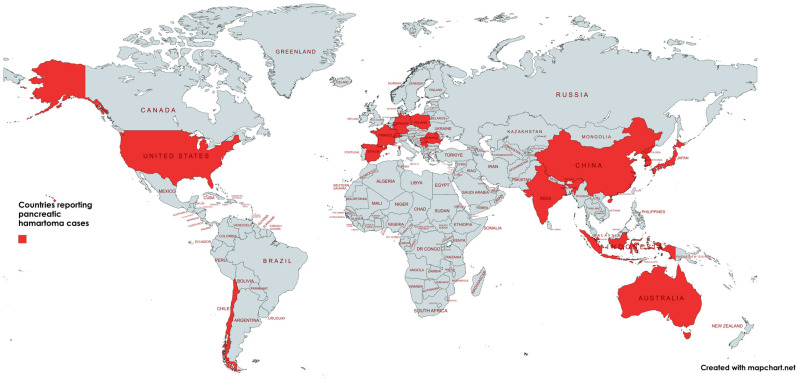
Countries reporting pancreatic hamartoma cases.

The characteristics of all 51 publications included in this systematic review are presented in detail in Table 1, Table 2, Table 3 and Table 4. A total of 77 cases were identified in the literature, comprising 68 cases in adults and 9 cases in neonates and children. The included studies were published between 1983 and 2024. Among all reported cases of PH, the highest number was from Japan (n = 15), followed by the United States (n = 10) and the Republic of Korea (n = 7). The geographical distribution of reported cases is shown in Figure 6. More than half of PH cases were males (52.9%), and the mean age was 59.5 ± 12.9 years, ranging from 25 to 78 years in the adult population. More than half of the patients were asymptomatic (57.4%), had pancreatic hamartoma located in the head (52.9%), body (19.1%), and tail (16.2%). The most common provisional diagnoses based on clinical and radiographic findings were neuroendocrine tumor (NET) (32.7%), unspecified pancreatic tumor (9.6%), and pancreatic cancer (9.6%). Regarding treatment modalities, the most frequently employed interventions included pylorus-preserving pancreaticoduodenectomy (PD) (18.5%), standard PD (18.5%), non-specified surgical resection (18.5%), and distal pancreatectomy (14.8%). Notably, the Whipple procedure was performed in 11.1% of all confirmed cases of pancreatic hamartoma. In Table 1 and Table 2, characteristics of studies with the adult and child and newborn population included in the systematic review are presented in detail.

Histological characteristics of pancreatic hamartoma cases of the adult population included in the systematic review are presented in Table 3. The most frequent pattern observed pathologically was solid (64.7%) and solid/cystic (35.3%). Spindle cell cellularity was predominantly low (46.2%). Additionally, Langerhans islets were absent in 91.8% of the reported cases. Immunohistochemical features of pancreatic hamartoma cases included in the systematic review are presented in detail in Table S1.

Median lesion size was 29 mm (IQR: 20–50). Mass effect was seen in 32% and biliary obstruction in 6.5%. Most lesions were well-defined (97%) and commonly showed solid-cystic or heterogeneous appearance. On MRI, they were typically low on T1 (76%) and high on T2 (89%). FDG-PET showed no uptake in 82% of cases. Enhancement patterns and signal intensity varied across imaging modalities (Table 4).

All available data from published case reports were systematically extracted and entered into a database to link radiological characteristics with pathological findings. Cases that were provisionally diagnosed as neuroendocrine tumors (NETs) more often showed low intensity on T1-weighted MRI (*p* = 0.015) (Table 5).

**Table 1:** Characteristics of studies with adult population included in the systematic review.

Author	Year	Country	Gender/Age	Presentation	Duration of Symptoms (Months)	Site	Size (cm)	Provisional Diagnosis	Treatment	Post-Op Complications	Follow-Up and Outcome
Izibicki et al. [17]	1994	Germany	M/25	Abdominal pain, nausea	/	H	10.6	/	/	/	/
Wu et al. [18]	1998	USA	M/39	Transient prandial midepigastric pain and a 15 kg weight loss over the past year.	10 years	H	8.0 × 8.0 × 6.0	/	Whipple	/	Recovered well since surgery without recurrence of pain after 9 mo
McFaul et al. [19]	2004	Switzerland	M/29	Heartburn, vomiting and intermittent right upper quadrant pain associated with a 15 kg weight loss	13 mo	H	1.0	Chronic pancreatitis and a pancreatic neoplasm; calcification also suggested a non-functioning islet cell neoplasm.	Pylorus-preserving PD	/	Alive and well after 2 years
			M/62	Intermittent abdominal pain associated with vomiting 1–2 h after eating and a 25 kg-weight loss	2 years	H	3.5	/	Kausch-Whipple procedure	/	Alive and well after 3 mo
Pauser et al. [5]	2005	Germany	F/36	Epigastric pain	1.0	H	7.0	Pseudotumor	Whipple	No	Relapse-free after 15 mo
			F/55	Abdominal pain	1 mo	N	2.7	Pseudotumor	Distal pancreatectomy and splenectomy	No	Relapse-free after 23 mo
Pauser et al. [6]	2005	Germany	M/51	Asymptomatic	/	T	3.0	/	Surgery	No	Relapse-free after 2 years
			M/54	Slight abdominal discomfort	/	B	2.0	/	Left-sided pancreatic resection	No	Relapse-free after 4 years
Nagata et al. [1]	2007	Japan	F/58	Asymptomatic	/	B	1.9	Pancreatic endocrine tumor	Distal pancreatectomy	No	Relapse-free after 6 mo
Samplean et al. [20]	2009	Romania	M/46	Abdominal pain	9 mo	H	8 × 6	/	Cephalic duodenopancreatectomy	/	/
Durczynski et al. [9]	2010	Poland	M/69	Asymptomatic	/	B	3.0	/	Central pancreatic resection with Roux-en-Y pancreaticojejunostomy to the distal pancreatic remnant	Yes	Relapse-free after 55 mo
Kawakami et al. [8]	2012	Japan	F/78	Asymptomatic	/	H	1.4	Pancreatic cancer	PD	/	Relapse-free after 30 mo
Kim et al. [21]	2012	Korea	F/52	Postprandial abdominal discomfort	1 mo	H	2.2 × 1.4	SPN or SCN	Pylorus-preserving PD	No	Relapse-free after 10 mo
Kwon et al. [22]	2012	Korea	F/41	Asymptomatic	/	H	2 × 1	NET or SPN	Pylorus preserving PD	No	/
Yamaguchi et al. [7]	2013	Japan	F/78	Asymptomatic	/	H	1.7	Pancreatic cancer	Surgical resection	/	Relapse-free after 32 mo
			F/61	Abdominal pain	/	H	4.0	SPN	Surgical resection	/	Relapse-free after 7 mo
			F/71	Asymptomatic	/	B and T	5.0	Cystic tumor	Surgical resection	/	Relapse-free after 68 mo
			F/58	Asymptomatic	/	B	2.0	NET	Surgical resection	/	Relapse-free after 6 mo
			F/59	Abdominal pain	/	T	1.0	NET	Surgical resection	/	Relapse-free after 10 mo
			M/53	Abdominal pain	/	H	8.0	SPN	Surgical resection	/	Relapse-free after 9 mo
			M/53	Asymptomatic	/	H	2.5	Mass-formatting pancreatitis	Surgical resection	/	Relapse-free after 12 mo
Addeo et al. [10]	2013	France	F/61	Asymptomatic	/	B	2.7 × 2.3 × 2.1	/	A robotic distal pancreatectomy with a splenectomy	/	/
Inoue et al. [23]	2014	Japan	M/65	Obstructive jaundice	/	H	4.0	/	PD	/	Relapse-free after 3 years
Shahbaz et al. [24]	2015	USA	F/62	Abdominal pain	3–4 mo	H	1.4	Pancreatic tumor	Whipple	/	/
Zhang et al. [25]	2016	China	F/53	Abdominal pain, anorexia, 2 kg weight loss	2 mo	H	2.3 × 1.5 × 1.5	/	Formal PD	No	No recurrence after 55 mo
Murakami et al. [26]	2016	Japan	F/62	Pancreatitis attacks	30 years	H	7.0 × 5.7 × 4.0	Pancreatitis	Subtotal stomach-preserving PD	No	Pancreatitis has not recurred since surgery
Matsushita et al. [27]	2016	Japan	M/68	Asymptomatic	/	U	4.0 × 4.0 × 2.8	Benign tumor such as lipoma, dermoid cyst or the other rare benign tumor	Pyloric preserving PD	No	No recurrence after 50 mo
Han et al. [14]	2017	Republic of Korea	F/35	Hypoglycemia	/	T	1	NET	Distal pancreatectomy	/	/
Nahm et al. [13]	2017	Australia	F/42	Abdominal pain	3 mo	N	2.8	SPN	Central pancreatectomy	/	No recurrence after 8 mo
Nagano et al. [28]	2017	Japan	F/72	Asymptomatic	/	H	2.0	/	Subtotal pyloric preserving PD	/	No recurrence afer 36 mo
Tanaka et al. [29]	2018	Japan	M/54	Asymptomatic	/	T	3.6	IPMN, SCN, malignant tumor	Distal pancreatectomy	/	/
			M/74	Asymptomatic	/	H	5.0	IPMN	PD	/	/
			M/67	Asymptomatic	/	T	6.5	Liposarcoma	Distal pancreatectomy	/	/
Shin et al. [30]	2019	Republic of Korea	F/54	Asymptomatic	/	H	2.2	SPN or NET	Robot-assisted pylorus-preserving PD	No	No evidence of local tumor recurrence or distant metastasis after 6 mo
Dasaraju et al. [31]	2020	USA	M/74	Asymptomatic	/	U	2.3	NET	PD	/	/
Zhou et al. [32]	2020	China	M/73	Abdominal pain	/	H	4 × 3.5	Pancreatic liposarcoma	PD	No	No sign of local recurrence after 3 mo
Katayama et al. [12]	2020	Japan	M/78	Asymptomatic	/	T	2.9 × 2.3	NET	Whipple	No	Disease-free after 34 mo
Cui et al. [33]	2020	China	F/57	Asymptomatic	/	U	2.5	NET	Whipple	/	Disease-free after 34 mo
			M/69	Asymptomatic	/	H	1.5	/	Whipple	/	Disease-free after 44 mo
Toyama et al. [11]	2020	Japan	M/53	Asymptomatic	/	H	3.7	Mixed solid and cystic type SCN, NET with cystic degeneration, IPMC, SPN, and slow flow vascular malformation (the so-called hemangioma)	PD	/	/
Yang et al. [34]	2021	Republic of Korea	M/68	Asymptomatic	/	H	1.8	NET	Pylorus-preserving PD	/	/
Jha et al. [35]	2021	India	M/68	Progressively enlarging lump	7 mo	B and T	19 × 18	/	Distal pancreatectomy with splenectomy	/	/
Noguchi et al. [36]	2021	Japan	F/70	Asymptomatic	/	B	1.9 × 1.6 × 1.4	Nonfunctional pancreatic NET	Surgical resection	/	/
Ahn et al. [37]	2021	Republic of Korea	M/68	Asymptomatic	/	H	1.8	NET	Pylorus-preserving PD	/	No recurrence or metastasis after 6 mo
Woo et al. [38]	2022	Japan	F/49	Asymptomatic	/	B	1.3 × 0.9	NEN and SPN	Laparoscopic distal pancreatectomy	/	/
Santana et al. [39]	2022	Spain	M/41	Asymptomatic	/	B	1.8	Mucinous tumor	Distal pancreatectomy	/	Alive after 5 mo
Tanigawa et al. [40]	2022	Japan	F/78	Asymptomatic	/	H	1.7	Pancreatic cancer	/	/	No recurrence after 98 mo
			F/71	Asymptomatic	/	B	5.0	Cystic tumor	/	/	/
			M/66	Asymptomatic	/	H	1.5	Pancreatic cancer	/	/	/
			F/58	Asymptomatic	/	B	2.0	PanNET	/	/	No recurrence after 6 mo
			M/65	Weight loss	/	H	4.0	Acinar cell carcinoma	/	/	No recurrence after 10 mo
			F/67	Asymptomatic	/	B	2.5	Pancreatic tumor	/	/	No recurrence after 6 mo
			M/75	Asymptomatic	/	T	1.0	Pancreatic cancer	/	/	No recurrence after 37 mo
			F/75	Asymptomatic	/	T	0.8	PanNET	/	/	No recurrence after 3 mo
			F/63	Asymptomatic	/	B	1.9	Paraganglioma	/	/	No recurrence after 48 mo
			F/59	Abdominal pain	/	T	1.0	PanNET	/	/	No recurrence after 9 mo
			M/53	Abdominal pain	/	H	8.0	SPN	/	/	No recurrence after 15 mo
			M/53	Asymptomatic	/	H	2.5	Mass forming pancreatitis	/	/	No recurrence after 16 mo
			M/68	Abdominal pain	/	H	4.0	Dermoid cyst	/	/	No recurrence after 132 mo
Tee et al. [41]	2022	Singapore	M/Middle age	Asymptomatic	/	B	13	Pancreatic lipoma and well-differentiated liposarcoma	Surgical resection	Yes	Well after 3 mo
Kim et al. [2]	2023	Republic of Korea	F/57	Asymptomatic	/	H	3.2	Primary hyperaldosteronism	Pylorus-preserving PD	No	No sign of recurrence after 6 mo
Jeo et al. [42]	2023	Indonesia	M/39	Jaundice	7 days	All pancreas	Multiple cysts ranging ±0.5–5	von-Hippel-Lindau disease	Total PD	Yes	Well after 6 mo
Shintaku et al. [3]	2023	Japan	F/68	Asymptomatic	/	H	1.8	NET	Subtotal stomach-preserving PD	No	Relapse-free after 9 mo
Das et al. [43]	2024	USA	M/73	Abdominal pain	/	T	2.3	/	Laparoscopic distal pancreatectomy and splenectomy	/	Patient alive and well after 5 years
			F/68	Abdominal pain	/	H	2.7	/	PD	/	Patient alive and well after 6 years
			M/73	Abdominal pain	/	T	0.6	/	Pancreatectomy	/	Patient alive and well after 6 years
Liu et al. [44]	2024	China	M/63	Abdominal distension with abdominal pain	15days	H	2.1	NET	PD	/	No sign of recurrence after 16 mo
Wan et al. [45]	2024	China	M/33	Asymptomatic	/	H	6 × 4	Clinical benign tumor	Laparoscopic enucleation	Yes	Relapse-free after 11 mo
Present case	2021	Serbia	F/37	Abdominal pain	Several years	T	3.5 × 3 × 2	Mucinous cystic neoplasm, mucinous cystadenoma	Distal pancreatectomy with splenectomy	No	Alive and well after 4 years

F, female; M, male; mo, months; head, H; tail, T; PD, pancreaticoduodenectomy; mo, months; w, weeks; y, years.

**Table 2:** Characteristics of studies with population of newborns and children included in the systematic review.

Author	Year	Country	Gender/Age	Site	Size (cm)	PH	Treatment	Follow-Up and Outcome
Burt et al. [46]	1983	USA	F/34 w	Diffuse	11.5	Solid and cystic	PD and splenectomy	Alive at 3 mo
Flaherthy et al. [47]	1992	USA	F/20 mo	H	9	Solid and cystic	Local resection	Alive at 9 mo
Sepulveda et al. [48]	2000	Chile	M/27 w	Diffuse	12	Large multicystic	Excision of the tumor and partial duodenectomy	Symptom-free at the age of 1 y
Thrall et al. [49]	2007	USA	M/3 y	H	3	Multicystic adenomatoid	PD	/
Sueyoshi et al. [50]	2009	Japan	M/14 mo	T	14	Multicystic adenomatoid	Local resection	Alive at 26 mo
Delgado et al. [51]	2017	USA	F/33 w	/	1.2	Cysts	/	Trisomy 18, autopsy case, alive 1 h
Shah et al. [52]	2017	USA	F/8 mo	/	14.5 × 11 × 5.5	Solid and cystic	Resection	Well after 3 mo
Hosfield et al. [53]	2019	USA	M/4 years	H	9.5	Multicystic adenomatoid	Whipple	Well after 3 mo
Varlas et al. [54]	2022	Romania	F/36 weeks	Diffuse	11	Multicystic adenomatoid	Local resection	Alive at 11 mo

F, female; M, male; mo, months; head, H; tail, T; PD, pancreaticoduodenectomy; mo, months; w, weeks; y, years.

**Table 3:** Histological characteristics of pancreatic hamartoma cases included in the systematic review.

Author	Year	Pathologic Type	Provisional Diagnosis	Spindle Cells Cellularity	Langerhans Islets	Peripheral Nerves	Elastic Fibers *
Wu et al. [18]	1998	/	Mucinous cystic tumor Suspicion of malignancy	/	/	/	/
McFaul et al. [19]	2004	S	Chronic pancreatitis, pancreatic neoplasm, islet cell neoplasm	/	/	/	/
		S		/	/	/	/
Pauser et al. [5]	2005	S/C	/	High	No	/	/
		S/C	/	Low	No	/	/
Pauser et al. [6]	2005	S	/	/	No	/	/
		S	/	/	No	/	/
Nagata et al. [1]	2007	S	Pancreatic endocrine tumor	Low	No	/	/
Sampelean et al. [20]	2009	/, myoepithelial	/	/	/	/	/
Durczynski et al. [9]	2011	S	/	Low	Yes	/	/
Kawakami et al. [8]	2012	S	/	Low	No	/	/
Kim et al. [21]	2012	S/C	SPN, SCN				
Kwon et al. [22]	2012	S, myoepithelial	/	/	/	/	/
Yamaguchi et al. [7]	2013	S	Pancreatic cancer	Moderate	No	No	No
		S	SPN	Low	No	No	No
		S/C	Cystic tumor	High	No	No	No
		S	NET	Moderate	No	No	No
		S	NET	Moderate	No	No	No
		S/C	SPN	Moderate	No	No	No
		S/C	Mass-forming pancreatitis	Low	No	No	No
Addeo et al. [10]	2014	S	Suspicion of malignancy	/	/	/	/
Inoue et al. [23]	2014	S	/	Low	/	/	/
Shasbaz et al. [24]	2015	S	Pancreatic carcinoma	/	Yes	/	/
Zhang et al. [25]	2016	S	/	/	/	/	/
Murakami et al. [26]	2016	S, myoepithelial	/	/	/	/	/
Matsushita et al. [27]	2016	S/C, lipomatous	Suspicion of malignancy	/	/	/	/
Han et al. [14]	2017	S/C	NET	/	Present	/	/
Nahm et al. [13]	2017	S/C, lipomatous	SPN	/	No	No	No
Nagano et al. [28]	2017	S	Suspicion of malignancy	/	No	/	/
Tanaka et al. [29]	2018	S, lipomatous	SCN, IPMN, malignant tumor with lipoid degeneration	/	No	No	No
		S, lipomatous	Lipoma	/	No	No	No
		S, lipomatous	Liposarcoma	/	No	No	No
Shin et al. [30]	2019	S	SPN, NET	/	/	/	/
Dasaraju et al. [31]	2020	S	Suspicion of malignancy	/	/	/	/
Zhou et al. [32]	2020	S, lipomatous	Pancreatic liposarcoma	/	No	No	/
Katayama et al. [12]	2020	S, lipomatous	NET, SPN	/	No	No	No
Cui et al. [33]	2020	S/C	NET	/	/	/	/
		S	Suspicion of malignancy	/	/	/	/
Toyama et al. [11]	2020	S/C	Mixed solid and cystic type SCN, NET, IPMN, SPN	/	No	No	/
Yang et al. [34]	2021	S	NET	/	/	/	/
Ahn et al. [37]	2021	S	NET	/	No	No	No
Noguchi et al. [36]	2021	S	NET	/	No	No	/
Woo et al. [38]	2022	S	Hypovascular NET, SPN	/	No	No	/
Santana et al. [39]	2022	S/C	NET	/	/	/	/
Tanigawa et al. [40]	2022	S	Pancreatic cancer	High	No	No	No
		S/C	Cystic tumor	High	No	No	No
		S	Pancreatic cancer	High	No	No	No
		S	NET	High	No	No	No
		S	Acinar cell carcinoma	High	No	No	No
		S	Pancreatic tumor	High	No	No	No
		S	Pancreatic cancer	Low	No	No	No
		S	NET	High	No	No	No
		S	Paraganglioma	Low	No	No	No
		S	NET	High	No	No	No
		S/C	SPN	Low	No	No	No
		S/C	Mass-forming pancreatitis	Low	No	No	No
		S/C, lipomatous	Dermoid cyst	Low	No	No	No
Tee at al. [41]	2022	S, lipomatous	Pancreatic lipoma, liposarcoma	/	No	No	No
Kim et al. [2]	2023	S/C	NET	/	/	/	/
Jeo et al. [42]	2023	S/C	Existing von-Hippel- Lindau disease	/	Yes	/	/
Shintaku et al. [3]	2023	S	NET, MNP, SPN	/	No	No	No
Das et al. [43]	2024	S/C	IPMN	/	No	/	/
		S/C	IPMN	/	No	/	/
		S/C	IPMN	/	/	/	/
Liu et al. [44]	2024	S	NET	/	/	/	/
Wan et al. [45]	2024	S/C, lipomatous	Teratoma	/	/	/	/
Present case	2025	S/C	/	Low	No	Yes	/

Elastic fibers * surrounding pancreatic ducts stained with Elastica von Gieson; S—solid; S/C—solid and cystic; CgA—chromogranin A; NET—neuroendocrine tumor; IPMN—intraductal papillary mucinous neoplasm; SPN—solid pseudopapillary neoplasm; PB—pancreatoblastoma; MNP—mucinous cystic neoplasm, SCN—serous cystic neoplasm; ACC—acinar cell carcinoma.

**Table 4:** Radiologic Features of Pancreatic Hamartoma.

Characteristic	n/N (%)
Size mm *	29 (20, 50)
Mass Effect	11/34 (32%)
Biliary Obstruction	2/31 (6.5%)
NECT	
fat density	3/18 (17%)
hypodense	6/18 (33%)
isodense	3/18 (17%)
mixed density	6/18 (33%)
CT+C A	
hyperdense	10/24 (42%)
hypodense	8/24 (33%)
isodense	3/24 (13%)
peripheral hyperdense	3/24 (13%)
CT+C PVP	
hyperdense	8/16 (50%)
hypodense	2/16 (13%)
isodense	2/16 (13%)
peripheral hyperdense	4/16 (25%)
CT+C DP	
hyperdense	7/8 (88%)
isodense	1/8 (13%)
Calcification	3/23 (13%)
P duct dil.	4/28 (14%)
Biopsy	8/24 (33%)
Demarcation	
ill	1/36 (2.8%)
well	35/36 (97%)
Inside	
cystic	5/35 (14%)
heterogenous	8/35 (23%)
homogenous	1/35 (2.9%)
lipomatous	5/35 (14%)
multicystic	5/35 (14%)
solid	1/35 (2.9%)
solid-cystic	10/35 (29%)
T1WI	
high intensity	2/17 (12%)
isointensity	2/17 (12%)
low intensity	13/17 (76%)
T2WI	
high intensity	17/19 (89%)
isointensity	2/19 (11%)
T1+C	
delayed enhancement	2/11 (18%)
enhancement	8/11 (73%)
hypoenhancing	1/11 (9.1%)
DWI	
high signal	3/12 (25%)
isointense	3/12 (25%)
marginal	4/12 (33%)
no signal	2/12 (17%)
Fat	2/7 (29%)
FDG-PET	
high accumulation	1/11 (9.1%)
low accumulation	1/11 (9.1%)
no accumulation	9/11 (82%)
Description of size change	
enlarged	5/6 (83%)
stable	1/6 (17%)

* Median (IQR).

## 4. Discussion

To the best of our knowledge, this is the first systematic review dedicated to synthesizing all reported cases of pancreatic hamartoma, an exceptionally rare benign pancreatic tumor. Owing to the limited number of published cases, this comprehensive analysis represents an important step toward clarifying its clinical, radiological, and histopathological characteristics. By systematically collating and examining the existing evidence, this review provides a consolidated reference point for clinicians and researchers while identifying recurrent patterns that may facilitate earlier recognition and more accurate distinction from malignant pancreatic neoplasms.

Clinically, pancreatic hamartomas are often silent or manifest with non-specific symptoms, which contributes significantly to their diagnostic complexity. In most instances, these lesions are detected incidentally during imaging performed for unrelated reasons, such as malignancy surveillance or evaluation of vague abdominal discomfort. In our analysis, 57.4% of patients were asymptomatic at the time of diagnosis, and among symptomatic individuals, the most common complaint was vague or intermittent abdominal pain. Symptomatic presentation was more frequent among older adults, who had a significantly higher mean age than asymptomatic patients (63.1 ± 11.3 years vs. 54.9 ± 13.5 years, *p* = 0.009). These findings are consistent with previous reports, which emphasize the absence of pathognomonic clinical features in pancreatic hamartoma [39,44].

Radiologically, pancreatic hamartomas are frequently misinterpreted as malignant tumors due to their rarity and the lack of well-established imaging criteria. They lack distinctive features on cross-sectional imaging modalities such as CT or MRI, and their appearance often overlaps with that of NETs, mucinous cystic neoplasms, IPMNs, and pancreatic adenocarcinoma. In our review, 32.7% of cases were initially classified as NETs, and approximately 20% were suspected to represent pancreatic cancer or other malignant entities. This considerable overlap often leads to radical surgical interventions, including pancreaticoduodenectomy and distal pancreatectomy, to exclude malignancy and obtain a definitive diagnosis.

Although the number of cases with detailed imaging descriptions remains limited, certain patterns have emerged. Han et al. reported that pancreatic hamartomas may appear solid or solid-cystic on CT or MRI, depending on their composition [14]. Smaller lesions tend to be solid, whereas larger or mixed tumors may exhibit cystic components. A key distinguishing feature described in several cases is progressive, delayed enhancement on post-contrast MRI, likely reflecting the fibrotic stroma characteristic of these lesions. This enhancement pattern may aid in differentiating pancreatic hamartomas from hypervascular tumors such as NETs, which typically show rapid arterial enhancement and less delayed retention. Furthermore, pancreatic hamartomas generally lack diffusion restriction on DWI and show no abnormal FDG uptake on PET, further supporting their benign nature [14].

Our findings are in line with these observations. On MRI, pancreatic hamartomas typically demonstrated low signal intensity on T1-weighted images (76%) and high signal intensity on T2-weighted images (89%), with FDG-PET showing no uptake in 82% of cases. Notably, lesions initially misdiagnosed as NETs were more likely to display low T1 signal intensity. In contrast to NETs, which commonly exhibit high signal on DWI, pancreatic hamartomas showed moderate or no diffusion restriction. This novel observation may provide radiologists with an additional clue during preoperative evaluation that pancreatic hamartoma should be included in the differential when evaluating pancreatic lesions with these characteristic imaging patterns, particularly in cases where malignant tumors are being considered. Han et al. similarly emphasized that a lesion demonstrating progressive enhancement without diffusion restriction should prompt consideration of pancreatic hamartoma [14]. Although not pathognomonic, these imaging features are critical for raising suspicion and potentially avoiding misclassification.

Histopathologically, pancreatic hamartomas are well-circumscribed lesions composed of mature acini and ducts arranged in a distorted architecture, lacking well-formed Langerhans islets and elastic fibers within the ductal walls. The amount of fibrous stroma varies, contributing to their characteristic microscopic appearance. Recognizing this constellation of features is essential, particularly in limited biopsy specimens where diagnostic confusion with other pancreatic entities can arise [3]. In our review, the predominant pathological pattern was solid (64.7%) or solid–cystic (35.3%), with low spindle cell cellularity in 46.2% of cases. Langerhans islets were absent in 91.8% of reported specimens. The benign nature of pancreatic hamartoma is supported by a low Ki-67 proliferative index, consistently reported across both solid and cystic forms of the lesion [5,6,25,29], which was also confirmed in our case. Even in solid variants, the Ki-67 indices remained low compared with malignant pancreatic neoplasms, reinforcing the benign and indolent nature of these tumors. Recognizing this association may assist clinicians in diagnostic interpretation and support a conservative management approach when malignancy has been reasonably excluded.

Fine-needle aspiration or core biopsy often yields scant material composed of normal ductal cells, making definitive preoperative diagnosis challenging [8]. However, advances in endoscopic ultrasound-guided biopsy and the use of new-generation needles combined with immunohistochemistry have significantly improved diagnostic accuracy [55]. As reported by Shintaku et al. [3], even postoperative EUS-FNA evaluation may be challenging, as the small sample size can result in nonspecific findings such as focal pancreatic atrophy and fibrosis. Nevertheless, the presence of mature acinar cells and ductal structures embedded in fibrous stroma, in the absence of islets, may suggest pancreatic hamartoma and should prompt consideration of this entity in the differential diagnosis. In cases where imaging and EUS-FNA findings raise suspicion of a benign lesion, careful radiologic follow-up or repeat sampling may be considered to avoid unnecessary surgery. However, given the rarity of the disease and the limitations of tissue sampling, definitive diagnosis often remains postoperative.

Immunohistochemically, stromal cell expression of CD34 and CD117 is characteristic of but not specific to pancreatic hamartoma and can aid in distinguishing it from other pancreatic lesions [5,6,7,10,12,23]. In cases lacking stromal cell CD34 and CD117 expression, as in our case, the coexistence of ductal and neural elements, absence of cytologic atypia, and a low Ki-67 proliferation index collectively support the diagnosis of pancreatic hamartoma and confirm its benign character. However, CD117 positivity may lead to misinterpretation as gastrointestinal stromal tumor, underscoring the importance of contextual histopathological evaluation. CD34 expression in stromal cells supports the diagnosis, and these markers, when interpreted carefully, can strengthen diagnostic confidence and reduce unnecessary surgery [40].

Careful use of cytological and histological assessment can enable accurate preoperative diagnosis and help avoid radical surgical procedures, which carry significant morbidity. Increased awareness of the subtle but distinctive imaging and histopathological features of pancreatic hamartomas is therefore essential to improve preoperative recognition. As Nahm et al. aptly described, these lesions may mimic the appearance of more aggressive tumors despite their benign nature [13]. While surgical resection is often ultimately required to secure a definitive diagnosis, benign and low-grade malignant lesions must be considered in the preoperative differential. Unlike pancreatic cancer, pancreatic hamartomas are associated with excellent long-term outcomes. When surgery is indicated, minimally invasive, parenchyma-sparing approaches that preserve gastrointestinal and pancreatic function are most appropriate, reflecting the indolent biological behavior of these tumors [29].

### Strengths and Limitations

This study represents the most comprehensive synthesis to date of all published cases of pancreatic hamartoma, integrating clinical, radiological, and histopathological findings to identify consistent diagnostic patterns. Additionally, by combining a newly documented case with systematic evidence, it provides novel insights into distinguishing imaging features, particularly the diagnostic value of T1-weighted MRI, and normal DWI patterns, which have direct clinical implications for improving preoperative diagnosis and reducing unnecessary radical surgery. The primary limitation of this study is that EUS-guided fine-needle aspiration (EUS-FNA) was not performed in our case, which could have provided additional preoperative diagnostic information and potentially avoided surgery. Instead, an ex tempore (intraoperative) biopsy was carried out. However, to date, preoperative diagnosis based on EUS-FNA specimens has not been reliably established. The systematic review is based solely on case reports and small case series, which are inherently prone to bias due to their descriptive nature and lack of standardized methodology. Publication bias is also likely, as rare or diagnostically challenging cases are more frequently reported. Additionally, substantial heterogeneity exists across the included studies, reflecting differences in reporting quality, diagnostic workup, imaging techniques, and pathological classification over several decades, resulting in missing or inconsistently reported data. Finally, preoperative diagnostic approaches varied widely, and tissue sampling was often unavailable, limiting conclusions regarding preoperative diagnosis.

## 5. Conclusions

This systematic review provides the most comprehensive synthesis to date of the clinical, radiological, and histopathological features of pancreatic hamartoma, a rare benign pancreatic tumor. Key findings include low T1-weighted signal intensity, and absence of diffusion restriction with isointense signal on DWI in lesions initially misdiagnosed as NETs. This association is based on a small subset of patients and should be interpreted cautiously. Further studies with larger cohorts are needed to determine whether this feature reliably distinguishes NETs from hamartomas. Clinicians should consider these radiographic characteristics, in conjunction with EUS-guided fine-needle aspiration and immunohistochemistry, when evaluating pancreatic masses. Incorporating these findings into diagnostic algorithms can support a provisional diagnosis of pancreatic hamartoma and help avoid unnecessary radical surgery, promoting more conservative and individualized management strategies.

## Figures and Tables

**Table 5:** Provisional diagnosis by T1-weighted MRI signal intensity of all included cases.

T1W1	Provisional Diagnosis		*p*-Value
	Other (n = 6)	NET (n = 8)	
High-intensity	2 (33.3)	0 (0.0)	0.015
Isointensity	2 (33.3)	0 (0.0)	
Low-intensity	2 (33.3)	8 (100.0)	

## Data Availability

The original contributions presented in this study are included in the article. Further inquiries can be directed to the corresponding authors.

## Supplementary Materials

The following supporting information can be downloaded at: https://www.mdpi.com/article/10.3390/jcm15010136/s1. Search Strategy; Table S1: Immunohistochemical features of pancreatic hamartoma cases included in the systematic review; PRISMA checklist [56]

## References

[1] Nagata S., Yamaguchi K., Inoue T., Yamaguchi H., Ito T., Gibo J., Tanaka M., Tsuneyoshi M. (2007). Solid pancreatic hamartoma. Pathol. Int..

[2] Kim J.H., Yoon S.H., Choi J.A., Kwak J.H., Kim M., Kim S.H. (2023). Pathologically confirmed pancreatic hamartoma after surgical resection with an aldosterone-producing adrenal tumor: A case report. J. Surg. Case Rep..

[3] Shintaku H., Gokita K., Oshima T., Suzuki K., Fujii K., Sugaya N., Tomii S., Onishi I., Shimada K., Ohashi K. (2023). Pancreatic hamartoma: Possibility of a preoperative diagnosis via endoscopic ultrasound–guided fine-needle aspiration biopsy. Diagn. Cytopathol..

[4] Anthony P.P., Faber R.G., Russell R.C. (1977). Pseudotumours of the pancreas. Br. Med. J..

[5] Pauser U., Kosmahl M., Krušlin B., Klimstra D.S., Klöppel G. (2005). Pancreatic Solid and Cystic Hamartoma in Adults: Characterization of a New Tumorous Lesion. Am. J. Surg. Pathol..

[6] Pauser U., Da Silva M.T.S., Placke J., Klimstra D.S., Klöppel G. (2005). Cellular hamartoma resembling gastrointestinal stromal tumor: A solid tumor of the pancreas expressing c-kit (CD117). Mod. Pathol..

[7] Yamaguchi H., Aishima S., Oda Y., Mizukami H., Tajiri T., Yamada S., Tasaki T., Yamakita K., Imai K., Kawakami F. (2013). Distinctive Histopathologic Findings of Pancreatic Hamartomas Suggesting Their “Hamartomatous” Nature: A Study of 9 Cases. Am. J. Surg. Pathol..

[8] Kawakami F. (2012). Multiple solid pancreatic hamartomas: A case report and review of the literature. World J. Gastrointest. Oncol..

[9] Durczynski A., Wiszniewski M., Olejniczak W., Polkowski M., Sporny S., Strzelczyk J. (2011). Asymptomatic solid pancreatic hamartoma. Arch. Med. Sci..

[10] Addeo P., Tudor G., Oussoultzoglou E., Averous G., Bachellier P. (2014). Pancreatic hamartoma. Surgery.

[11] Toyama K., Matsusaka Y., Okuda S., Miura E., Kubota N., Masugi Y., Kitago M., Hori S., Yokose T., Shinoda M. (2020). A case of pancreatic hamartoma with characteristic radiological findings: Radiological-pathological correlation. Abdom. Radiol..

[12] Katayama H., Azuma K., Koneri K., Murakami M., Hirono Y., Hatta S., Imamura Y., Goi T. (2020). A typical case of resected pancreatic hamartoma: A case report and literature review on imaging and pathology. Surg. Case Rep..

[13] Nahm C.B., Najdawi F., Reagh J., Kaufman A., Mittal A., Gill A.J., Samra J.S. (2019). Pancreatic hamartoma: A sheep in wolf’s clothing. ANZ J. Surg..

[14] Han Y.E., Park B.J., Sung D.J., Kim M.J., Han N.Y., Sim K.C., Cho S.B., Kim J.Y. (2018). Computed tomography and magnetic resonance imaging findings of pancreatic hamartoma: A case report and literature review. Clin. Imaging.

[15] Riley D.S., Barber M.S., Kienle G.S., Aronson J.K., von Schoen-Angerer T., Tugwell P., Kiene H., Helfand M., Altman D.G., Sox H. (2017). CARE guidelines for case reports: Explanation and elaboration document. J. Clin. Epidemiol..

[16] Liberati A., Altman D.G., Tetzlaff J., Mulrow C., Gøtzsche P.C., Ioannidis J.P., Clarke M., Devereaux P.J., Kleijnen J., Moher D. (2009). The PRISMA statement for reporting systematic reviews and meta-analyses of studies that evaluate healthcare interventions: Explanation and elaboration. BMJ.

[17] Izbicki J.R., Knoefel W.T., Müller-Höcker J., Mandelkow H.K. (1994). Pancreatic hamartoma: A benign tumor of the pancreas. Am. J. Gastroenterol..

[18] Wu S.S., Vargas H.I., French S.W. (1998). Pancreatic hamartoma with Langerhans cell histiocytosis in a draining lymph node. Histopathology.

[19] McFaul C., Vitone L., Campbell F., Azadeh B., Hughes M., Garvey C., Ghaneh P., Neoptolemos J., Longnecker D.S. (2004). Pancreatic hamartoma. Pancreatology.

[20] Sampelean D., Adam M., Muntean V., Hanescu B., Domsa I. (2009). Pancreatic hamartoma and SAPHO syndrome: A case report. J. Gastrointestin Liver Dis..

[21] Kim H.H., Cho C.K., Hur Y.H., Koh Y.S., Kim J.C., Kim H.J., Lee J.H. (2012). Pancreatic hamartoma diagnosed after surgical resection. J. Korean Surg. Soc..

[22] Kwon W., Kim S.-W., Lee K.B., Jang J.-Y., Park J.W., Han I.W., Kang M.J. (2012). Myoepithelial hamartoma as a solitary mass in the pancreatic parenchyma: The first case report. Korean J. Hepato-Biliary-Pancreat. Surg..

[23] Inoue H., Tameda M., Yamada R., Tano S., Kasturahara M., Hamada Y., Tanaka K., Horiki N., Takei Y. (2014). Pancreatic hamartoma: A rare cause of obstructive jaundice. Endoscopy.

[24] Shahbaz O., Mitesh B.P. (2015). Pancreatic Hamartoma: A Mimicker of Pancreatic Adenocarcinoma: 252. Am. J. Gastroenterol..

[25] Zhang J., Wang H., Tang X., Jiang Q., Wang C. (2016). Pancreatic hamartoma, a rare benign disease of the pancreas: A case report. Oncol. Lett..

[26] Murakami T., Yamazaki M., Yamazaki K., Matsuo K., Hirano A., Hiroshima Y., Kawaguchi D., Ishida Y., Suzuki Y., Sugiyama M. (2016). A distinctive myoepithelial hamartoma of the pancreas histologically confirmed in the mother of a previously reported patient. Pancreatology.

[27] Matsushita D., Kurahara H., Mataki Y., Maemura K., Higashi M., Iino S., Sakoda M., Shinchi H., Ueno S., Natsugoe S. (2016). Pancreatic hamartoma: A case report and literature review. BMC Gastroenterol..

[28] Nagano H., Nakajo M., Fukukura Y., Kajiya Y., Tani A., Tanaka S., Toyota M., Niihara T., Kitazono M., Suenaga T. (2017). A small pancreatic hamartoma with an obstruction of the main pancreatic duct and avid FDG uptake mimicking a malignant pancreatic tumor: A systematic case review. BMC Gastroenterol..

[29] Tanaka M., Ushiku T., Ikemura M., Takazawa Y., Igari T., Shimizu M., Yamaguchi H., Fukushima N., Sakuma K., Arita J. (2018). Pancreatic Lipomatous Hamartoma: A Hitherto Unrecognized Variant. Am. J. Surg. Pathol..

[30] Shin D.H., Rho S.Y., Hwang H.K., Lee W.J., Kang C.M. (2019). A case of pancreatic hamartoma pathologically confirmed after robot-assisted pancreaticoduodenectomy. Ann. Hepato-Biliary-Pancreat. Surg..

[31] Dasaraju S., Liu S., Kelly R., Mneimneh W. (2020). A Case Of Pancreatic Hamartoma Mimicking Malignancy: An Uncommon Pitfall. Am. J. Clin. Pathol..

[32] Zhou B., Li G., Xu S., Zhan C., Zheng X., Yan S. (2020). Pancreatic lipomatous hamartoma mimicking other pancreatic tumor: A case report and literature review. Am. J. Transl. Res..

[33] Cui H., Lian Y., Chen F. (2020). Imaging findings for pancreatic Hamartoma: Two case reports and a review of the literature. BMC Gastroenterol..

[34] Yang J.D., Lee M.R., Ahn S.W., Yu H.C. (2021). Pancreatic solid harmatoma mimicking neuroendocrine tumor: Case report. Ann. Hepato-Biliary-Pancreat. Surg..

[35] Jha A.K. (2021). Vascular Hamartoma of the Pancreas. Am. J. Gastroenterol..

[36] Noguchi T., Ryozawa S., Mizuide M., Tanisaka Y., Fujita A., Ogawa T., Suzuki M., Katsuda H., Nagata K., Kawasaki T. (2021). Pancreatic Hamartoma Difficult to Diagnose Preoperatively. Intern. Med..

[37] Ahn A.R., Song J.S., Do Yang J., Moon W.S. (2021). Pancreatic hamartoma mimicking neuroendocrine tumor. Pathol. Int..

[38] Woo J., Haradome H., Adachi K., Iwai T., Nishizawa N., Murakumo Y., Kusano C., Kumamoto Y., Inoue Y., Ojiri H. (2022). A case of solid-type pancreatic hamartoma presenting high apparent diffusion coefficient value: Histopathological correlation and literature review. Abdom. Radiol..

[39] Santana Valenciano Á., Molina Villar J.M., Barranquero A.G., Sanjuanbenito Dehesa A., Fernández Cebrián J.M. (2022). Pancreatic hamartoma: A rare and benign cause of pancreatic incidentaloma. Cir. Esp. Engl. Ed..

[40] Tanigawa M., Koga Y., Naito Y., Yamaguchi H., Iwasaki T., Kohashi K., Ohike N., Hanada K., Higashi M., Komatsu M. (2022). Pancreatic hamartoma: Detection of harbouring *NAB2::STAT6* fusion gene. Histopathology.

[41] Tee C.L., Lin E.Y., Bundele M.M., Low J.K. (2022). Rare case of pancreatic lipomatous hamartoma. BMJ Case Rep..

[42] Jeo W.S., Razi K., Setiawan A., Hartono R.W. (2023). Total pancreatoduodenectomy for multiple pancreatic cysts in von Hippel-Lindau disease presenting as obstructive jaundice: A case report. Int. J. Surg. Case Rep..

[43] Das D., Gonzalez I.A., Yeh M.M., Wu T.T., Jain D. (2024). Ductal hamartoma of the pancreas: A clinicopathologic study. Hum. Pathol..

[44] Liu S., Yang L., Wu J., Lin X., Zhang Z. (2024). Imaging and histopathologic characteristics of typical pancreatic hamartoma: A case report and literature review. Front. Oncol..

[45] Wan D.-L., Tong R.-L., Tong X.-Y., Hu C., Ke Q.-H., Yang X., Wu J. (2024). Laparoscopic enucleation for pancreatic lipomatous hamartoma. Hepatobiliary Pancreat. Dis. Int..

[46] Burt T.B., Condon V.R., Matlak M.E. (1983). Fetal pancreatic hamartoma. Pediatr. Radiol..

[47] Flaherty M.J., Benjamin D.R. (1992). Multicystic pancreatic hamartoma: A distinctive lesion with immunohistochemical and ultrastructural study. Hum. Pathol..

[48] Sepulveda W., Carstens E., Sanchez J., Gutierrez J. (2000). Prenatal diagnosis of congenital pancreatic cyst: Case report and review of the literature. J. Ultrasound Med..

[49] Thrall M., Jessurun J., Stelow E.B., Adsay N.V., Vickers S.M., Whitson A.K., Saltzman D.A., Pambuccian S.E. (2008). Multicystic Adenomatoid Hamartoma of the Pancreas: A Hitherto Undescribed Pancreatic Tumor Occurring in a 3-Year-Old Boy. Pediatr. Dev. Pathol..

[50] Sueyoshi R., Okazaki T., Lane G.J., Arakawa A., Yao T., Yamataka A. (2013). Multicystic adenomatoid pancreatic hamartoma in a child: Case report and literature review. Int. J. Surg. Case Rep..

[51] Delgado P.I., Correa-Medina M., Rojas C.P. (2017). Pancreatic hamartoma in a premature Trisomy 18 female. Autops. Case Rep..

[52] Shah U., Goldstein A.M., Gee M.S., Deshpande V. (2017). Case 24-2017: An 8-Month-Old Girl with Fever and an Abdominal Mass. N. Engl. J. Med..

[53] Hosfield B.D., Grayson B.L., Nakeeb A., Albright E.A., Markel T.A. (2019). Multicystic adenomatoid hamartoma of the pancreas. J. Pediatr. Surg. Case Rep..

[54] Varlas V., Neagu O., Moga A., Bălănescu R., Bohiltea R., Vladareanu R., Balanescu L. (2022). Fetal Pancreatic Hamartoma Associated with Hepatoblastoma—An Unusual Tumor Association. Diagnostics.

[55] Crinò S.F., Bernardoni L., Manfrin E., Parisi A., Gabbrielli A. (2016). Endoscopic ultrasound features of pancreatic schwannoma. Endosc. Ultrasound.

[56] Page M.J., McKenzie J.E., Bossuyt P.M., Boutron I., Hoffmann T.C., Mulrow C.D., Shamseer L., Tetzlaff J.M., Akl E.J., Brennan S.E. (2021). The PRISMA 2020 statement: An updated guideline for reporting systematic reviews. BMJ.

