# Supplementary material for: Bridging Imaging and Pathohistology in Pancreatic Hamartoma: A Systematic Review of the Literature with an Integrated Case Report
Source: J Clin Med. 2025 Dec 24;15(1):136. doi: 10.3390/jcm15010136 (PMC12786915; doi:10.3390/jcm15010136)
Supplement: Supplementary file 1 [file jcm-15-00136-s001.zip › jcm-4007839-supplementary.pdf]

Search strategy

PubMed: (("pancreas"[MeSH Terms] OR "pancreas"[All Fields] OR "pancreatic"[All Fields] OR "pancreatitides"[All Fields] OR "pancreatitis"[MeSH Terms] OR "pancreatitis"[All Fields]) AND ("hamartoma"[MeSH Terms] OR "hamartoma"[All Fields] OR "hamartomas"[All Fields])) OR (("pancrea"[All Fields] OR "pancreas"[MeSH Terms] OR "pancreas"[All Fields]) AND ("hamartoma"[MeSH Terms] OR "hamartoma"[All Fields] OR "hamartomas"[All Fields]))

Scopus: ALL ("pancreatic hamartoma" OR "pancreas hamartoma")

Web of Science: (TS=(pancreatic hamartoma)) OR (TS=(pancreas hamartoma))

Table S1. Immunohistochemical features of pancreatic hamartoma cases included in the systematic review.

Author	Year	CK7	CK19	CD34	CD117	S-100	STAT6	bcl-2	Ki67	CgA	SYN	Other
Flaherty et al. [47]	1992									neg		pos: insulin, glucagon, somatostatin, pancreatic polypeptide
Wu et al. [18]	1988	/	/	/	/	/	/	/	/	/	/	/
Sepulveda et al. [48]	2000	/	/	/	/	/	/	/	/	/	/	/
McFaul et al. [19]	2004	/	/	/	/	/	/	/	/	/	/	/
		/	/	/	/	/	/	/	/	/	/	/
Pauser et al. [5]	2005			pos	pos				rare	pos		pos: trypsin, CK8, insulin<br>neg: glucagon, somatostatin, pancreatic polypeptide, SMA
				pos	pos				rare	pos		
Pauser et al. [6]	2005	/	/	pos	pos	/	/	/	rare	/	/	pos: Cam 5.2, trypsin, pancreatic polypeptide somatostatin, KiM1P, CK8<br>neg: insulin, glucagon
		/	/	pos	pos	/	/	/	rare	/	/	
Nagata et al. [1]	2007	/	/	pos	pos	neg	/	/	/	pos	/	pos: insulin, somatostatin, glucagon, amylase, trypsin, CAM5.2, AE1/AE3, EMA, neg: α-SMA, muscle actin
Thrall et al. [49]	2008	pos	/	pos	neg	/	/	neg	few	pos	/	pos: CK20, CDX2, insulin, somatostatin, gastrin, CD10, SMA<br>neg: MUC1, ER, PR, inhibin
Sampelean et al. [20]	2009	/	/	/	/	/	/	/	/	/	/	/
Durczynski et al. [9]	2011	/	/	pos	pos	pos	/	/	/	pos	/	pos: insulin, glucagon, somatostatin, amylase, desmin
Kwon et al. [22]	2012	/	/	neg	/	/	/	/	/	/	/	pos: SMA
Sueyoshi et al. [50]	2013	/	/	/	/	/	/	/	/	/	/	/
Kim et al. [21]	2012	/	/	pos	pos	/	/	/	neg	neg	pos	pos: CD56<br>neg: desmin, actin

Kawakami et al. [8]	2012	/	/	pos	pos	neg	/	neg				pos: CAM5.2, AE1/AE3, EMA, amlylase, trypsin, SMA, desmin
Addeo et al. [10]	2014	/	/	neg	neg	/	/	pos	/	/	/	/
Yamaguchi et al. [7]	2013	/	/	pos	neg	pos	/	/	/	neg	/	/
		/	/	pos	neg	pos	/	/	/	neg	/	/
		/	/	pos	pos	pos	/	/	/	neg	/	/
		/	/	pos	pos	pos	/	/	/	neg	/	/
		/	/	pos	pos	pos	/	/	/	neg	/	/
		/	/	pos	neg	pos	/	/	/	neg	/	/
		/	/	pos	pos	pos	/	/	/	neg	/	/
Inoue et al. [23]	2014	/	/	pos	neg	pos	/	/	/	/	/	/
Shasbaz et al. [24]	2015	/	/	/	/	/	/	/	/	/	/	/
Matsushita et al. [27]	2016	pos	pos	/	/	pos	/	/	few	/	/	neg: CK20
Murakami et al. [26]	2016	/	/	/	/	/	/	/	/	/	/	pos: SMA
Zhang et al. [25]	2016	/	neg	/	pos	pos	/	/	<1%	pos	pos	pos: α-1-antitrypsin, α-1-antichymotrypsin, CD56, PR<br>neg: p63, vimentin
Shah et al. [52]	2017	/	/	/	/	/	/	/	/	neg	/	pos: desmin<br>neg: ER, insulin, glucagon
Han et al. [14]	2017	/	/	pos	neg	/	/	/	/	/	/	/
Nagano et al. [28]	2017	/	/	/	/	/	/	/	/	neg	neg	pos: epithelial markers, GLUT-1, HK-II
Delgado et al. [51]	2017	/	/	/	/	/	/	/	/	/	/	/
Nahm et al. [13]	2017	/	/	pos	/	pos	/	/	/	neg	/	/
Tanaka et al. [29]	2018	/	/	pos	neg	neg	/	pos	<1%	/	/	pos: ER, HMGA2, CD10<br>neg: α-inhibin, PR, DOG-1, SMA, MUC1, MUC5AC, MUC6, CLDN18
		/	/	pos	neg	neg		pos	<1%	/	/	pos: ER, HMGA2, CD10

												neg: α-inhibin, PR, DOG-1, SMA, MUC1, MUC5AC, MUC6, CLDN18
		/	/	pos	neg	neg		neg	<1%	/	/	neg: α-inhibin, ER, PR, CD10, DOG-1, SMA, MUC1, MUC5AC, MUC6, CLDN18
Hosfield et al. [53]	2019	/	/	/	/	/	/	/	/	/	/	/
Shin et al. [30]	2019	/	/	neg	neg	/	/	/	/	/	/	/
Toyama et al. [11]	2020	/	/	/	/	/	/	/	/	/	/	/
Cui et al. [33]	2020	/	/	/	/	/	/	/	pos	/	/	pos: vimentin, SMA<br>neg: CD138, IgG4, IgG, CK (pan)
Zhou et al. [32]	2020	pos	pos	/	/	pos	/	/	/	/	/	neg: HMB45, Melan-A
Katayama et al. [12]	2020	pos	/	pos	pos	pos	/	pos	/	/	/	pos: ER, CK20
Dasaraju et al. [31]	2020	/	/	neg	neg	/	/	/	1%	neg	neg	neg: CD56
Noguchi et al. [36]	2021	/	/	/	/	/	/	/	/	neg	neg	/
Ahn et al. [37]	2021	pos	pos	pos	/	/	/	/	/	/	/	/
Tanigawa et al. [40]	2022	/	/	pos	neg	pos	neg	/	/	/	/	/
		/	/	pos	pos	pos	pos	/	/	/	/	/
		/	/	pos	neg	pos	neg	/	/	/	/	/
		/	/	pos	pos	pos	pos	/	/	/	/	/
		/	/	pos	neg	pos	pos	/	/	/	/	/
		/	/	pos	neg	pos	pos	/	/	/	/	/
		/	/	pos	pos	pos	pos	/	/	/	/	/
		/	/	pos	pos	pos	pos	/	/	/	/	/
		/	/	pos	neg	pos	neg	/	/	/	/	/
		/	/	pos	pos	pos	pos	/	/	/	/	/
		/	/	pos	neg	pos	neg	/	/	/	/	/
		/	/	pos	pos	pos	neg	/	/	/	/	/
		/	/	neg	neg	pos	neg	/	/	/	/	/
Tee at al. [41]	2022	/	/	/	/	/	/	/	/	/	/	neg: SMA, HMB45

Varlas et al. [54]	2022	pos	pos	/	/	/	/	/	15%	/	/	pos: CK8/18, CK20, CDX2
Woo et al. [38]	2022	/	/	pos	neg	/	/	/	/	neg	neg	pos: AE1AE3, EMA, CAM 5.2, CA19-9
Santana et al. [39]	2022	/	/	neg	/	neg	/	neg	/	neg	neg	pos: β-catenin<br>neg: IgG, IgG4, actin
Kim et al. [2]	2023	pos	pos	/	/	pos	/	pos	/	/	/	/
Jeo et al. [42]	2023	pos	pos	/	/	/	/	/	0.2%	pos	pos	neg: CEA
Shintaku et al. [3]	2023	/	/	pos	neg	/	neg	/	1-2%	neg	neg	neg: CD56, IgG4
Das et al. [43]	2024	pos	pos	/	/	/	/	/	/	/	/	pos: MUC-5AC<br>neg: MUC6, MUC2
		/	/	/	/	/	/	/	/	/	/	
		/	/	/	/	/	/	/	/	/	/	
Liu et al. [44]	2024	pos	pos	/	/	/	/	/	<5%	neg	neg	pos: β-catenin<br>neg: CD56
Wan et al. [45]	2024	pos	/	/	/	/	/	/		pos	pos	pos: MUC1, MUC6, MUC-5AC<br>neg: PR, ER
Present case	2025	pos	pos	neg	neg	neg	/	/	3%	/	/	pos: CK AE1/AE3, CAM5.2, MUC1, MUC5AC, vimentin, α-SMA<br>neg: CK20, mCEA, MUC4, desmin

CEA – carcinoembryonic antigen; CgA – chromogranin A; PR – progesterone receptor; ER – estrogen receptor; STAT6 - signal transducer and activator of transcription 6; SMA – smooth muscle actin; GLUT-1 - glucose transporter type I; HK-II – hexokinase II
